# Natriuretic peptide activation of extracellular regulated kinase 1/2 (ERK1/2) pathway by particulate guanylyl cyclases in GH3 somatolactotropes

**DOI:** 10.1007/s00441-017-2624-x

**Published:** 2017-04-27

**Authors:** Kim C. Jonas, Timothy Melrose, Iain R. Thompson, Gary F. Baxter, Victoria J. Lipscomb, Stijn J. Niessen, Charlotte Lawson, Craig A. McArdle, Mark S. Roberson, Imelda M. McGonnell, Caroline P. Wheeler-Jones, Robert C. Fowkes

**Affiliations:** 1 Endocrine Signalling Group, Department of Comparative Biomedical Sciences, Royal Veterinary College, University of London, 4 Royal College Street, London, NW1 0TU UK; 2 Comparative Biomedical Sciences, Royal Veterinary College, University of London, London, NW1 0TU UK; 3 Division of Pharmacology, The Welsh School of Pharmacy, Cardiff University, King Edward VII Avenue, Cardiff, CF10 3NB UK; 4 Clinical Sciences & Services, Hawkshead Lane, North Mymms, Hatfield, Hertfordshire, UK; 5 Laboratories for Integrative Neuroscience and Endocrinology, Department of Clinical Sciences at South Bristol, University of Bristol, Whitson Street, Bristol, BS13NY UK; 6 Department of Biomedical Sciences, College of Veterinary Medicine, Cornell University, Ithaca, NY 14853 USA

**Keywords:** Natriuretic, cGMP, Guanylyl cyclase, Pituitary, ERK1/2

## Abstract

The natriuretic peptides, Atrial-, B-type and C-type natriuretric peptides (ANP, BNP, CNP), are regulators of many endocrine tissues and exert their effects predominantly through the activation of their specific guanylyl cyclase receptors (GC-A and GC-B) to generate cGMP. Whereas cGMP-independent signalling has been reported in response to natriuretic peptides, this is mediated via either the clearance receptor (Npr-C) or a renal-specific NPR-Bi isoform, which both lack intrinsic guanylyl cyclase activity. Here, we report evidence of GC-B-dependent cGMP-independent signalling in pituitary GH3 cells. Stimulation of GH3 cells with CNP resulted in a rapid and sustained enhancement of ERK1/2 phosphorylation (P-ERK1/2), an effect that was not mimicked by dibutryl-cGMP. Furthermore, CNP-stimulated P-ERK1/2 occurred at concentrations below that required for cGMP accumulation. The effect of CNP on P-ERK1/2 was sensitive to pharmacological blockade of MEK (U0126) and Src kinases (PP2). Silencing of the GC-B1 and GC-B2 splice variants of the GC-B receptor by using targeted short interfering RNAs completely blocked the CNP effects on P-ERK1/2. CNP failed to alter GH3 cell proliferation or cell cycle distribution but caused a concentration-dependent increase in the activity of the human glycoprotein α-subunit promoter (αGSU) in a MEK-dependent manner. Finally, CNP also activated the p38 and JNK MAPK pathways in GH3 cells. These findings reveal an additional mechanism of GC-B signalling and suggest additional biological roles for CNP in its target tissues.

**Electronic supplementary material:**

The online version of this article (doi:10.1007/s00441-017-2624-x) contains supplementary material, which is available to authorized users.

## Introduction

The mammalian family of particulate guanylyl cyclases currently comprises six identified members (GC-A to -F) that act as receptors for the natriuretic peptides, heat-stable enterotoxins and odour molecules and as phototransducers (Potter et al. 2006; Garbers et al. 2006). These receptors and their orthologues mediate a diverse range of functions across many species. The natriuretic peptides, Atrial-, B-type and C-type natriuretic peptides (ANP, BNP and CNP, respectively), exert the vast majority of their effects via either the GC-A (*Npr1*, ANP-specific) or GC-B (*Npr2*, CNP-specific) receptors (Potter et al. 2006; Garbers et al. 2006) by mechanisms that involve the intrinsic guanylyl cyclase activity of these receptors, leading directly to cGMP generation. Although some non-cGMP-mediated effects of natriuretic peptides have been reported, these are thought to be mediated via the non-guanylyl cyclase, NPR-C (*Npr3*) receptor (also referred to as the clearance receptor; Anand-Srivastava 2005). Investigation of GC-B knock-out mice has suggested roles for GC-B signalling in bone development, female reproductive function and pituitary growth hormone expression (Chusho et al. 2001; Tamura et al. 2004). However, in many GC-B-expressing tissues, the regulation of local cGMP concentrations remains the only biological function identified to date (Fowkes and McArdle 2000).

The anterior pituitary gland expresses high tissue concentrations of the GC-B ligand, CNP, plus all three natriuretic peptide receptors (Fowkes and McArdle 2000; Komatsu et al. 1991; Grandclément et al. 1995). Our previous studies revealed that CNP potently stimulates cGMP accumulation in rodent pituitary cell lines and primary rat pituitary cultures (Fowkes et al. 2000; Shimekake et al. 1994; Thompson et al. 2009, 2014) and some studies have indicated a function for GC-B and cGMP in mediating pituitary growth hormone secretion (Shimekake et al. 1994; Hartt et al. 1995). Furthermore, we have demonstrated the expression of CNP and GC-B in fetal, normal adult and adenomatous human pituitary tissue, including those from acromegalic patients (Thompson et al. 2012). However, the effects of CNP on somatotrope proliferation and associated signalling pathways have not been established.

Several groups have investigated the potent anti-proliferative effects of the natriuretic peptides and CNP, in particular, by mechanisms involving the GC-B/cGMP/protein kinase G (PKG) inhibition of the mitogen-activated protein kinase (MAPK) pathway (Hagiwara et al. 1996; Kaneki et al. 2008). Recently, a combination of CNP and the phosphodiesterase 5a inhibitor, Sildenafil, has been shown to inhibit MAPK signalling and cell proliferation of rhabdomyosarcoma cells (Zenitani et al. 2016). Interestingly, this same combination of treatments potentiates the growth of melanoma cells in a cGMP/PKG/MAPK-dependent manner (Dhayade et al. 2016). In our current study, we seek to investigate the potential role of CNP in pituitary MAPK signalling. We demonstrate that CNP potently stimulates the phosphorylation of extracellular regulated kinase 1/2 (ERK1/2) in pituitary GH3 somatolactotrope cells at concentrations at which cGMP levels are not affected. These effects appear to be cGMP-independent but GC-B–dependent, as determined by silencing of the GC-B receptor, indicating an alternative mechanism by which members of the particulate guanylyl cyclase family may signal.

## Materials and methods

### Cell culture and treatments

GH3, αT3–1 and LβT2 cells were grown and maintained in Dulbecco’s modified Eagle’s medium (DMEM; Sigma, Poole, UK) containing penicillin/streptomycin and 10% fetal calf serum (FCS; Invitrogen, Paisley, UK) at 37 °C, 5% (v/v) CO_2_ in air as previously described (Thompson et al. 2012, 2014). Cells were plated at a density of 1 × 10^6^ cells/well for Western blotting and RNA extraction and at 3 × 10^5^ cells/well for reporter gene assays and cGMP determinations. CNP, ANP, forskolin, db-cGMP, BAPTA-AM, GF109203X, genistein, H89, herbimycin A, nifedipine, protein phosphatase 2 (PP2), PP3, PI3K (phosphatidyl inositol 3-kinase) inhibitor (LY294006) and U0126 were obtained from Calbiochem (Merck Chemicals, UK) or Sigma (Sigma-Aldrich, Poole, UK). All compounds, except for nifedipine, were stored aliquoted as stock solutions at -20 °C.

### Immunoblotting

Cells were serum-starved overnight prior to drug treatment. Following stimulation, cell lysates were harvested in lysis buffer (0.5 M TRIS pH 6.8, 10% (v/v) glycerol, 1% (w/v) SDS) containing phosphatase inhibitor cocktails 1 and 2 (Sigma, Dorset, UK). Protein lysates were sonicated, boiled at 95 °C for 5 min, resolved by using 10% SDS-polyacrylamide gel electrophoresis, transferred to polyvinylidene difluoride membrane and probed with the following: primary antibodies against P-P-ERK1/2, total ERK1/2, P-p90RSK, P-Elk-1, P-CREB, CREB, P-p38, total p38, P-JNK, total JNK (all from Cell Signalling, NEB, Hitchin, UK), GC-B (Santa Cruz, Calif., USA) and β-Actin (Abcam, Cambridge, UK). Appropriate horseradish peroxidase (HRP)-conjugated secondary antibodies were purchased from DAKO (Cambridge, UK). In some instances, scanning densitometric analysis was carried out by using a BioRad scanner and the Quantity One scanning densitometry software package (Bio-Rad Hertfordshire, UK). The density of the P-ERK1/2 and total ERK1/2 bands in each lane was quantified and the ratio of P-ERK1/2 to the corresponding total ERK1/2 lane was calculated.

### cGMP and cAMP enzyme immunoassay

Cell treatments were conducted in physiological saline solution (PSS: 127 mM NaCl, 1.8 mM CaCl_2_, 5 mM KCl, 2 mM MgCl_2_, 0.5 nM NaH_2_PO_4_, 5 mM NaHCO_3_, 10 mM glucose, 10 mM HEPES pH 7.4, 0.1% [w/v] bovine serum albumin) in the presence of 1 mM 3-isobutyl-1-methylxanthine (IBMX), as described previously (Thompson et al. 2012). Cell stimulations were terminated by addition of ice-cold absolute ethanol and cGMP or cAMP concentrations determined as instructed by the manufacturer (R&D Systems, Abingdon, UK).

### Transfections and RNA interference

For transfections, 3 × 10^5^ GH3 cells/well were plated in 24-well plates and transfected by using Lipofectin (Invitrogen) with 1.25 μg/well -517αLUC together with 0.5 μg/well BosβGal. Total protein extracts were assayed for reporter gene activity as described previously (Thompson et al. 2009). For RNA interference, pre-designed GC-B short interfering RNA (siRNA) or scrambled RNA control (Ambion Biosciences, Invitrogen) were introduced into GH3 cells by the lipofectin siRNAmax reverse-transfection method (Invitrogen). For MAPK phosphatase-1 (MKP1) over-expression studies, 5 μg/well MKP1 or pcDNA expression vectors were reverse-transfected into GH3 cells, prior to serum starvation and subsequent stimulation.

### Reverse transcription and polymerase chain reaction

For reverse transcription (RT), total RNA was extracted by using Tri-reagent (Sigma, Poole, UK) and subjected to DNase treatment (Qiagen, Crawley, UK) before the generation of first-strand cDNA (Applied Biosystems, UK). The polymerase chain reaction (PCR) was performed for a range of targets over 30 cycles with the following primers: *Npr1* (5′) AAGCTTATCTGGAGGAGAAGCGCA and (3′) TCAGCCTCGAGTGCTACATCCCCG; *Npr2* (5′) GGTACCAGCATATTGGACAACCTC and (3′) CAGGAGTCCAGGAGGTCTTTTTCG; *GC-B1/2* (5′) GAAGCTGATGCTGGAGAAGGAGC and (3′) GACAATACTCGGTGACAATGCAG; *GC-B1/3* (5′) ACGGGCGCATTGTGTATATCTGCGGC and TTATCACAGGATGGGTCGTCCAAGTCA; *Npr3* (5′) CCCTCCAAACAGTCACCCTA and (3′) CCATCCTTCTTGCTGTAGCC; *Rpl19* (5′) CTGAAGGTCAAAGGGAATGTG and (3′) GGACAGAGTCTTGATGATCTC.

### Cell proliferation assays

For all assays, GH3 cells were plated into 6-well plates in DMEM supplemented with 2% (v/v) FCS (control), 2% (v/v) FCS containing 100 nM CNP, or 10% (v/v) FCS (positive control) and grown for 48 h before being harvested. For cell counting, cells were harvested and cell numbers were determined by 0.4% (w/v) trypan blue exclusion methodology. For flow cytometry analysis, cells were harvested and re-suspended in 500 μl phosphate-buffered saline containing 0.1% (w/v) glucose, before being fixed in 70% (v/v) ice-cold ethanol, washed and re-suspended in a propidium iodide solution (69 μM propidium iodide, 38 mM sodium citrate, 20 mg/ml RNase pH 7.4) and incubated for 40 min at 37 °C. Cells were subjected to analysis by means of a Beckman Coulter EPICS XL-MCL flow cytometer and Beckman Coulter Expo 32 software, as described previously (Thompson et al. 2009). For tritiated thymidine incorporation, cells were incubated with 1 μCi/well tritiated thymidine for the last 6 h of the 48 h incubation period, before being harvested with 0.5 M NaOH/0.5% sodium dodecyl sulphate (SDS) and the addition of scintillation fluid. Samples were counted by using a Packard 1500 TriCarb Scintillation analyser with built-in software, as described previously (Thompson et al. 2009). Metabolic activity assays (MTT assay) were performed by using the Promega CellTiter 96 Aqueous One assay according to the manufacturer’s instructions (Promega, Southampton, UK). Briefly, 20 μl/well CellTiter 96 AQueous One Solution Reagent was added at the end of the 48 h incubation, before measurement of the absorbance at 490 nm on a Berthold Technologies Mithras LB940 plate reader with MicroWin-4.40-associated software.

### Data presentation and analyses

All experiments were performed a minimum of three times, with autoradiographs being representative of these experiments. For cGMP, cAMP, cell proliferation and luciferase activities, the data shown are normalised and represent the means ± SEM of at least three independent experiments, each performed in triplicate. Numerical data were subjected to analysis of variance (ANOVA) and were followed, where appropriate, by Bonferroni’s multiple comparisons test, accepting *P* < 0.05 as significant, with in-built equations in GraphPad Prism 7.0a (GraphPad, San Diego, Calif., USA).

## Results

### CNP and ANP effects on ERK1/2 phosphorylation in GH3 somatolactotropes

**Fig. 1:**
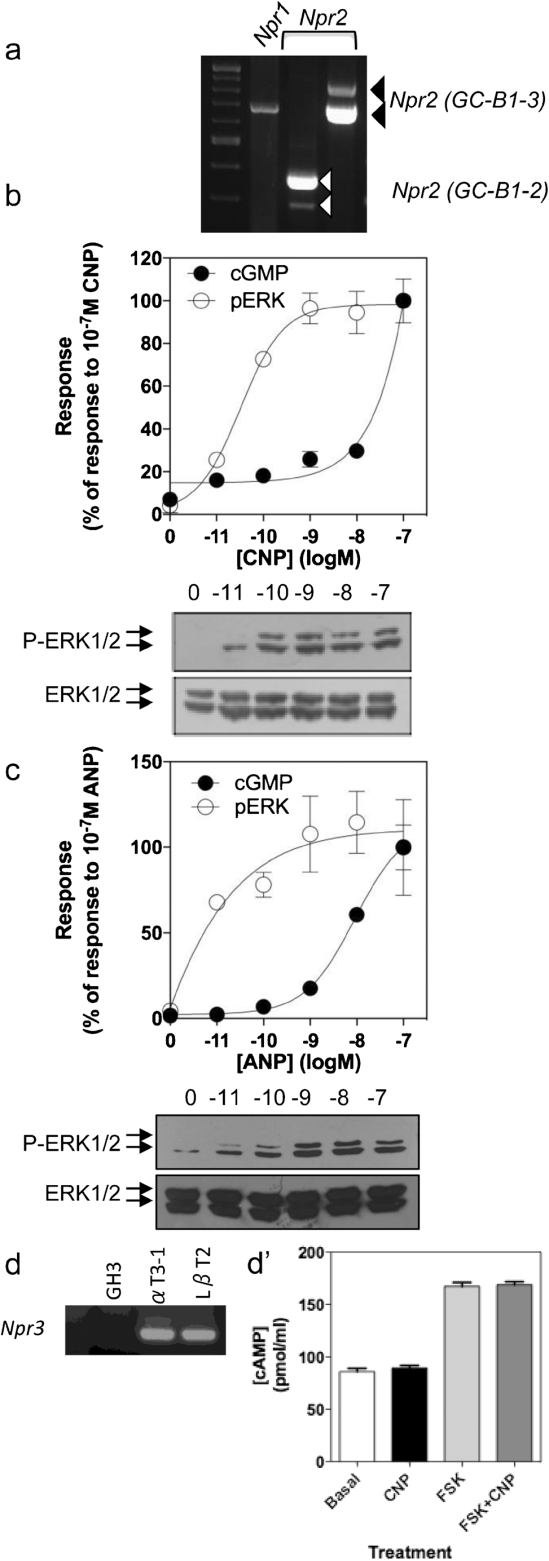
Natriuretic peptide effects on extracellular regulated kinase 1/2 (ERK1/2) phosphorylation and cGMP accumulation in GH3 cells. **a** Reverse transcription plus polymerase chain reaction (RT-PCR) analyses of GC-A (*Npr1*) and GC-B (*Npr2*) expression in GH3 cells. **b**, **c** Concentration-response studies of ERK1/2 phosphorylation (*P-ERK1/2*) or cGMP accumulation in response to (**b**) C-type natriuretric peptide (*CNP*) or (**c**) Atrial-type natriuretric peptide (*ANP*). In both instances, cells were stimulated for 15 min with the indicated concentrations of CNP prior to extraction of total proteins for Western blotting or total (intra- and extracellular) cGMP accumulation, as determined by cGMP enzyme immunoassay. Each autoradiograph is representative of at least three independent experiments; the scanning densitometry of ERK1/2 phosphorylation is pooled from at least three independent experiments. The cGMP data are expressed as the percentage of response to 10^−7^ M CNP or ANP for both ERK1/2 phosphorylation and cGMP accumulation. **d** RT-PCR analysis of *Npr3* expression in GH3 cells and mouse gonadotrope αT3–1 and LβT2 cells (30 cycles). **d’** GH3 cells were untreated (*Basal*) or treated with CNP (100 nM), forskolin (*FSK*, 10 μM) or both CNP and FSK for 15 min prior to the determination of total (intra- and extracellular) cAMP accumulation by cAMP enzyme immunoassay. Data shown are means ± SEM from three independent determinations

Initial RT-PCR analyses were performed to determine the particulate guanylyl cyclases that were expressed in GH3 cells. As shown (Fig. 1a), GH3 cells expressed *Npr1* (encoding GC-A) and all three splice variants of *Npr2* (encoding GC-B). Having established the presence of transcripts for both GC-A and GC-B receptors in GH3 cells, we next investigated whether these receptors were functional by measuring cGMP accumulation. GH3 cells were stimulated with a range of concentrations of CNP or ANP (from 10pM to 100 nM) in PSS containing 1 mM IBMX to inhibit phosphodiesterase activity. As shown (Fig. 1b, c), both CNP and ANP caused an increase in the total concentration of cGMP, indicating the presence of functional GC-B and GC-A receptors, as we and others have described previously (Shimekake et al. 1994; Fowkes et al. 2000; Thompson et al. 2014). To establish whether CNP and ANP could stimulate P-ERK1/2, we stimulated GH3 cells with the same concentration range of both peptides for 15 min, prior to the extraction of total protein and Western blotting for phosphorylated ERK1/2 (P-ERK1/2). Interestingly, not only did both peptides cause enhanced P-ERK1/2 but they did so at concentrations below those needed for cGMP accumulation, indicating that CNP and ANP can activate the cGMP and the ERK1/2 pathways in GH3 cells, albeit at differing efficacies. The similar nature of the P-ERK1/2 response to CNP and ANP suggested the involvement of a common receptor, such as the NPR-C clearance receptor. However, RT-PCR analysis revealed that GH3 cells failed to express transcripts for *Npr3* encoding NPR-C (Fig. 1d), in keeping with our previous studies (Thompson et al. 2014). As the inhibition of cAMP production has previously been indicated as evidence of NPR-C activity (Sciarretta et al. 2013), we examined the potential effect of CNP on forskolin (FSK)-stimulated cAMP accumulation in GH3 cells. As shown (Fig. 1d’), CNP failed to alter the FSK-stimulated cAMP increase, suggesting a lack of functional NPR-C receptors in these GH3 cells.

**Fig. 2:**
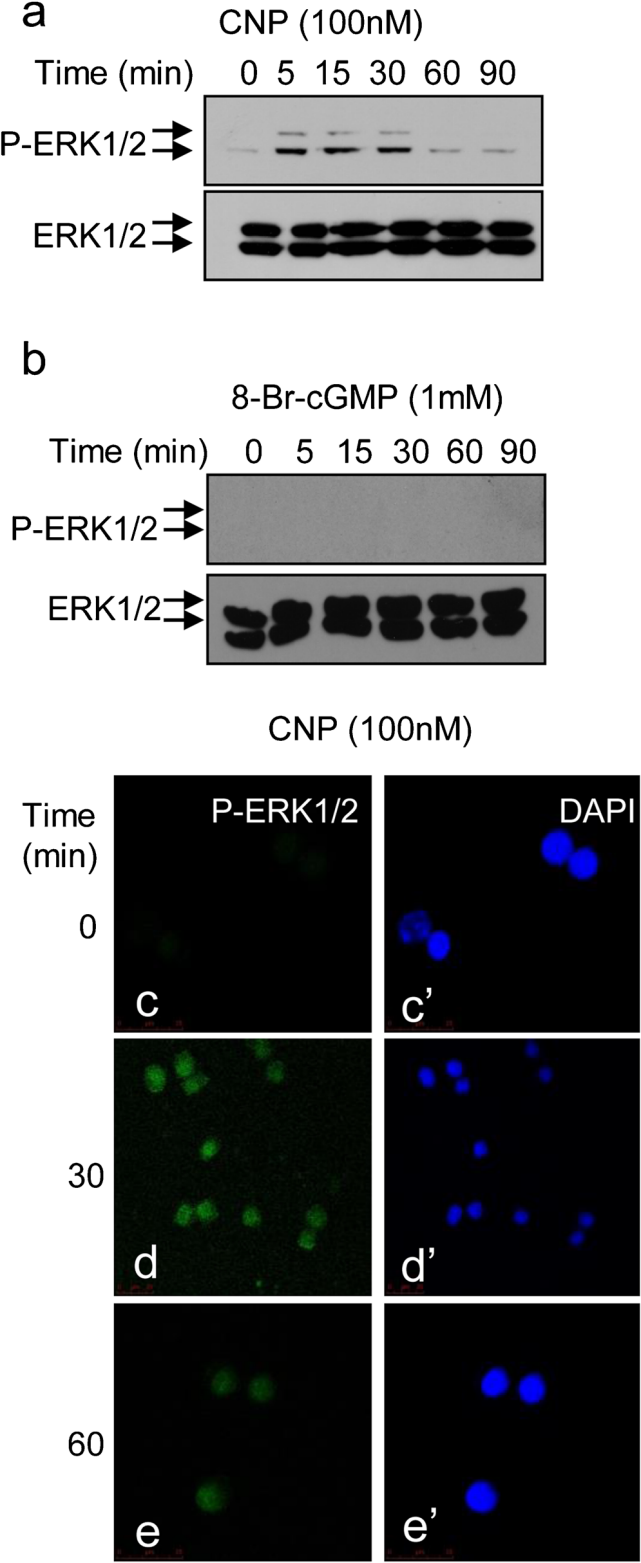
Time-course analysis of CNP and dibutryl-cGMP (db-cGMP) effects on ERK1/2 phosphorylation in GH3 cells. **a**, **b** GH3 cells were stimulated with (**a**) CNP (100 nM) or (**b**) db-cGMP (*8-Br-cGMP*; 1 mM) for up to 90 min, prior to extraction of total proteins and Western blotting for ERK1/2 phosphorylation. Each autoradiograph is representative of at least three independent experiments. **c–e’** GH3 cells were treated for up to 90 min with 100 nM CNP prior to being fixed and stained for phospho-ERK1/2 (Alexa-488, *green*) or nuclear co-stained (*DAPI* 4,6-diamidino-2-phenylindole, *blue*). Immunofluorescence was visualised using confocal microscopy. Images shown are a representative field of vision from at least three independent experiments

As previous studies have implicated CNP as the major natriuretic peptide of the anterior pituitary (McArdle et al. 1993; Fowkes et al. 2000; Thompson et al. 2009, 2014), we focused our subsequent experiments on the CNP/GC-B response in GH3 cells. To characterise the ERK1/2 response to CNP further, we examined the kinetics and localisation of P-ERK1/2 in GH3 cells. GH3 cells treated with 100 nM CNP showed a rapid enhancement in P-ERK1/2 within 5 min (Fig. 2a); this was sustained for at least 30 min. To establish whether the elevation of cGMP levels alone could mimic the P-ERK1/2 response, a similar time-course experiment was performed by using the cell permeable cGMP analogue, dibutryl-cGMP (db-cGMP); as shown (Fig. 2b), 1 mM db-cGMP failed to enhance P-ERK1/2 levels at any time point. As the sub-cellular location of P-ERK1/2 can influence its biological role, we used immunofluorescent microscopy to examine the spatial properties of CNP-stimulated P-ERK1/2. As shown (Fig. 2c–e), P-ERK1/2 was located in the nucleus of GH3 cells following treatment with 100 nM CNP for 30 mins and remained there for at least 60 min.

### GC-B dependence of CNP-stimulated P-ERK1/2 in GH3 somatolactotropes

**Fig. 3:**
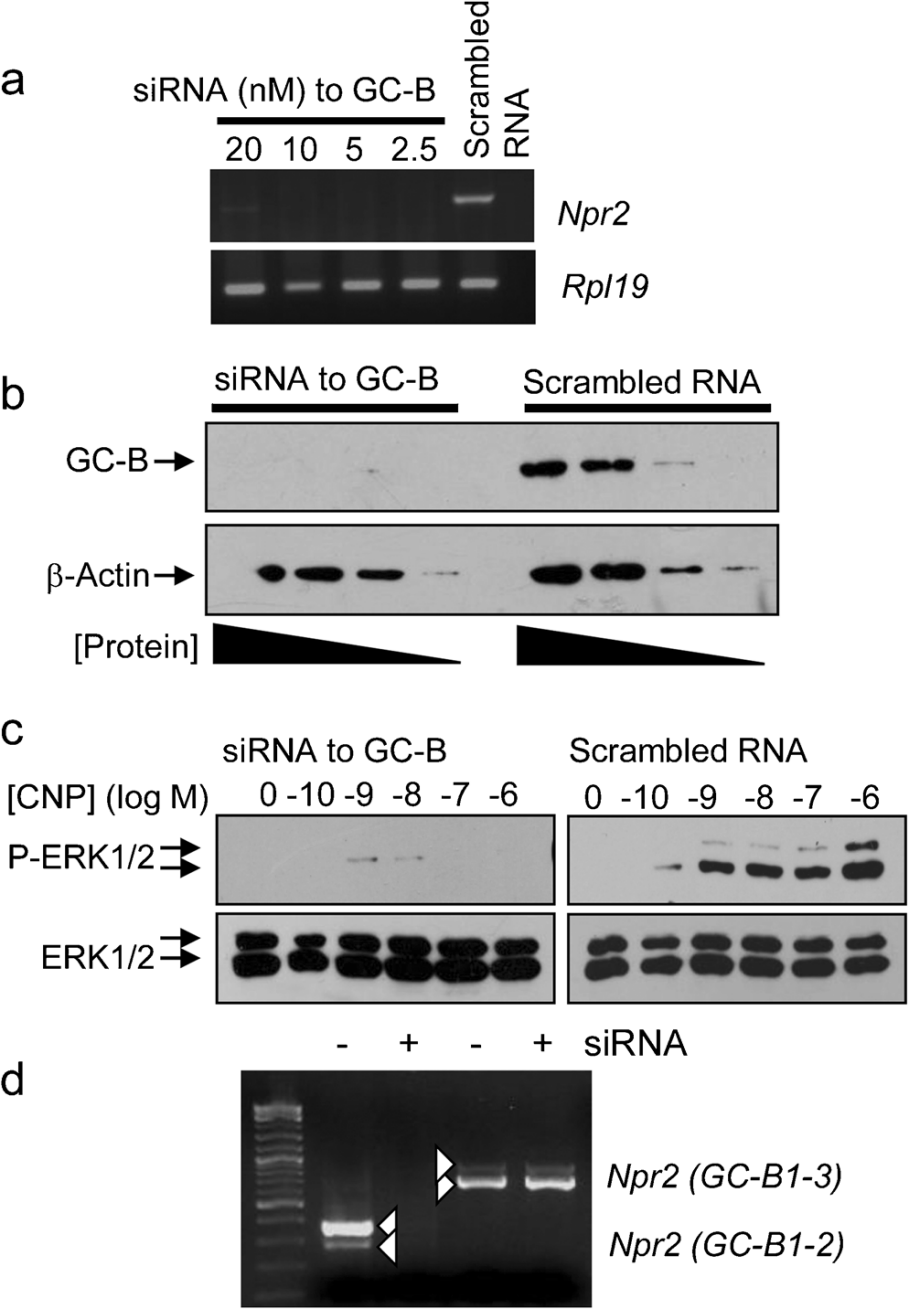
CNP stimulation of ERK1/2 phosphorylation is GC-B dependent. **a** RT-PCR analyses showing the optimisation of GC-B silencing by using short interfering RNA (*siRNA*). Total RNA was prepared from GH3 cells and reverse-transfected with the indicated concentrations of siRNA to GC-B or scrambled RNA control (20 nM) and harvested at 48 h post-transfection. **b** Western blotting to determine loss of GC-B protein in GC-B siRNA-treated GH3 cells (decreasing protein concentrations loaded per well). Total proteins were extracted at 48 h post-transfection and cell lysates were subjected to Western blot analyses for GC-B and then stripped and re-probed for β-Actin. **c** Western blotting analyses of GC-B siRNA-treated GH3 cells. GC-B siRNA or scrambled RNA-treated GH3 cells were stimulated with the indicated concentrations of CNP for 15 min. Cell lysates were subjected to Western blot analyses for phospho-ERK1/2 and then stripped and re-probed for total ERK1/2. **d** Specific silencing of GC-B1 and GC-B2 splice variant expression by using GC-B siRNA resulted in the loss of GC-B1 and GC-B2 (as determined by primers targeting the exon 20 to 22 region) but not GC-B1 and GC-B3 (as determined by primers targeting exon 5). In each case, the major band represents GC-B1. Each autoradiograph is representative of at least three independent experiments

Having excluded a possible role for the NPR-C receptor in mediating the CNP effects on P-ERK1/2, we next determined whether these effects were mediated via the GC-B receptor. Specific siRNA targeting of the GC-B receptor was performed in GH3 cells; RT-PCR for GC-B expression revealed the silencing of GC-B mRNA expression with the GC-B-specific siRNA but not with the scrambled RNA controls (Fig. 3a). Loss of GC-B protein in siRNA-treated GH3 cells was confirmed by Western blotting (Fig. 3b). Having confirmed the successful silencing of GC-B expression, we performed functional assays to determine the dependency of CNP-stimulated P-ERK1/2 on GC-B receptors. As shown, GC-B-silenced GH3 cells failed to respond to 100 nM CNP in terms of P-ERK1/2 (Fig. 3c, left), although these responses remained intact in scrambled RNA-treated GH3 cells (Fig. 3c, right). As the GC-B-specific siRNA oligonucleotides were known to target exons 21 and 22 in the *Npr2* gene (present in both GC-B1 and GC-B2 splice variants), we performed RT-PCR on siRNA-treated GH3 cells. As shown (Fig. 3d), siRNA-treated GH3 cells specifically lost the expression of GC-B1 and GC-B2 transcripts but not the truncated GC-B3 expression. Collectively, these data demonstrate the potential requirement for both GC-B isoforms in mediating CNP effects on P-ERK1/2 in GH3 somatolactotropes.

### Mechanisms of CNP-stimulated P-ERK1/2 in GH3 cells

As cGMP alone failed to mimic the effects of CNP on P-ERK1/2, we next examined putative signalling mechanisms to explain these effects of CNP on the ERK1/2 pathway. GH3 cells pre-treated with the MAPK kinase 1/2 (MEK1/2) inhibitor (U0126; 1-30 μM for 30 min) exhibited blunted P-ERK1/2 in response to CNP treatment (Fig. 4a, left). In similar studies, the Src-kinase-selective inhibitor (PP2; 1-30 μM for 30 min) concentration-dependently attenuated CNP-stimulated P-ERK1/2 (Fig. 4a, right). Interestingly, other receptor tyrosine kinase inhibitors (genistein, herbimycin A, PP3; 10 μM), all failed to affect CNP-stimulated P-ERK1/2 (Fig. 4b). As CNP can alter Ca^2+^ mobilisation, PI3K, protein kinase A (PKA) and PKC activity in a range of cells (Fowkes et al. 1999; Sun et al. 2006; Rose et al. 2007; Chen et al. 2014; De Jonge et al. 2014), we examined the requirement for these pathways in mediating the effects of CNP. GH3 cells pre-treated (1-30 μM for 30 min or 15 min) with BAPTA-AM, nifedipine, H89, GF109203X or PI3K inhibitor (LY294006), all failed to alter CNP effects on P-ERK1/2 (Supplemental Fig. 1). Having demonstrated the pharmacological blockade of CNP-stimulated P-ERK1/2 with U0126, we used the over-expression of the ERK1/2-associated phosphatase, MKP1, to disrupt CNP signalling. As shown (Fig. 4c), GH3 cells transiently transfected with 5 μg/well MKP-1 expression vector expressed MKP1 protein more abundantly than cells transfected with the empty pcDNA vector. The presence of overexpressed MKP1 protein led to an attenuation in CNP-stimulated P-ERK1/2 (Fig. 4c). Finally, in order to determine whether CNP-stimulated cGMP accumulation was dependent upon MEK activity, GH3 cells were pretreated for 30 min with 0 or 30 μM U0126, before being stimulated for 15 min with PSS containing 0 or 100 nM CNP in the presence of 1 mM IBMX. As shown (Fig. 4d), the presence of U0126 failed to affect significantly the CNP-stimulated cGMP accumulation (87.6 ± 8.8% cf. control response, *P* > 0.5). Collectively, these data suggest that CNP-stimulated P-ERK1/2 is reliant on MEK1/2 and Src kinase activity but is independent of Ca^2+^, PKA, PKC or PI3K activity.

**Figure:**
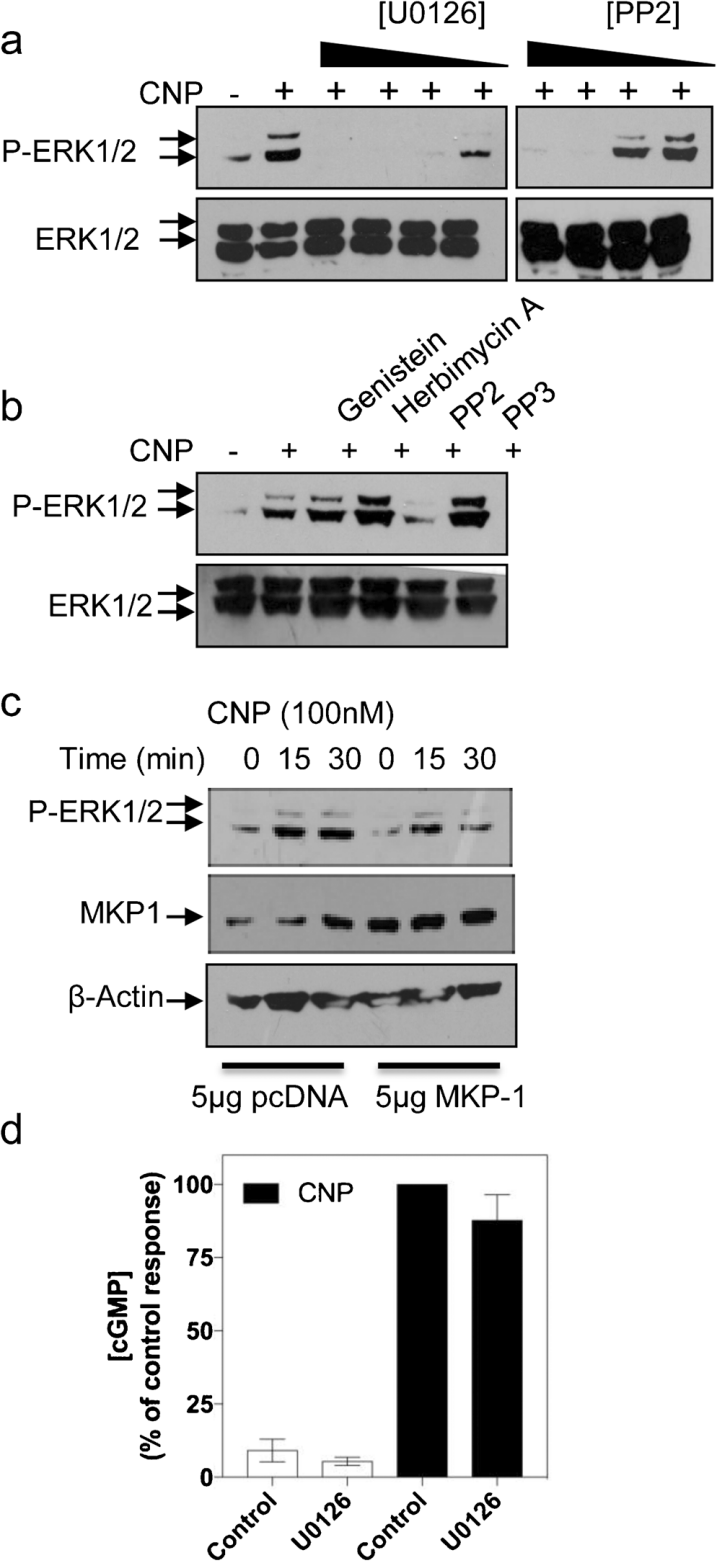

### Biological consequence of CNP-stimulated P-ERK1/2 in GH3 cells

Having established that the effects of CNP on P-ERK1/2 were independent of cGMP but dependent upon GC-B, we next investigated the potential biological consequence of CNP-stimulated P-ERK1/2. As ERK activity often regulates cell proliferation, we examined the role of CNP on cell proliferation (by using cell counting, thymidine incorporation and metabolic activity assays) and cell cycle distribution (by using flow cytometry). For each assay, GH3 cells were grown in 2% (v/v) FCS-containing media and treated with 0 or 100 nM CNP for 48 h, prior to being harvested. Cells grown in normal medium (containing 10% [v/v] FCS) acted as a positive control. In all cases, CNP failed to have an effect, whereas the 10% serum-treated cells all demonstrated a significantly increased cell number (by 160.3 ± 7.4%, *P* < 0.0001; Fig. 5a), increased thymidine incorporation (to 126.8 ± 4.5%, *P* < 0.0001; Fig. 5b) or increased metabolic activity (to 161.5 ± 5.1%, *P* < 0.0001; Fig. 5c). CNP also failed to alter significantly the cell-cycle distribution (Fig. 5d) in GH3 cells, although the presence of 10% (v/v) FCS significantly enhanced S-phase and G2/M distribution (to 62.9 ± 0.5% and 19.7 ± 0.5%, respectively; *P* < 0.0001), while reducing G0/G1 phase distribution (to 17.4 ± 0.1%, *P* < 0.0001).

**Figure:**
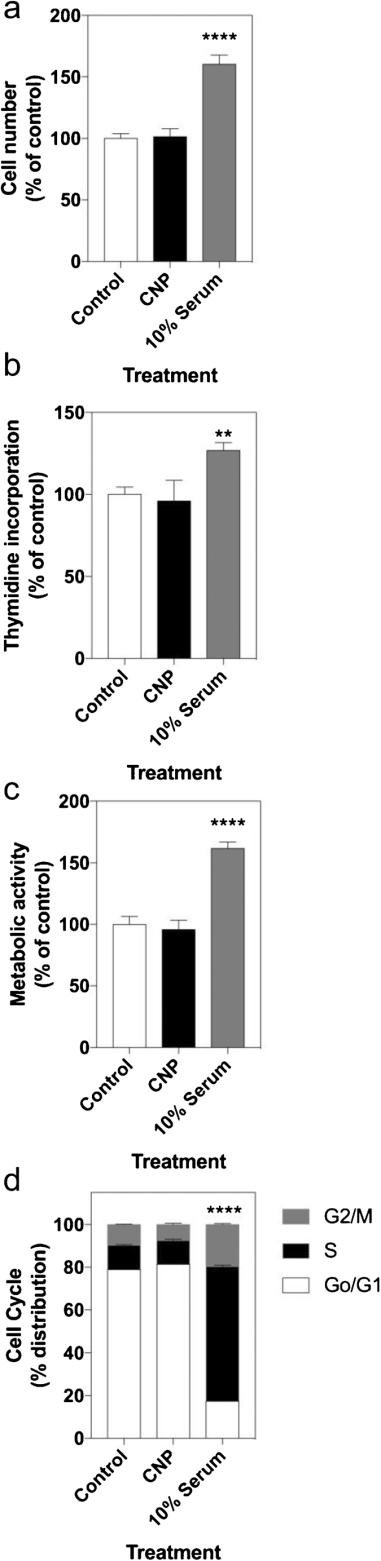

**Fig. 6:**
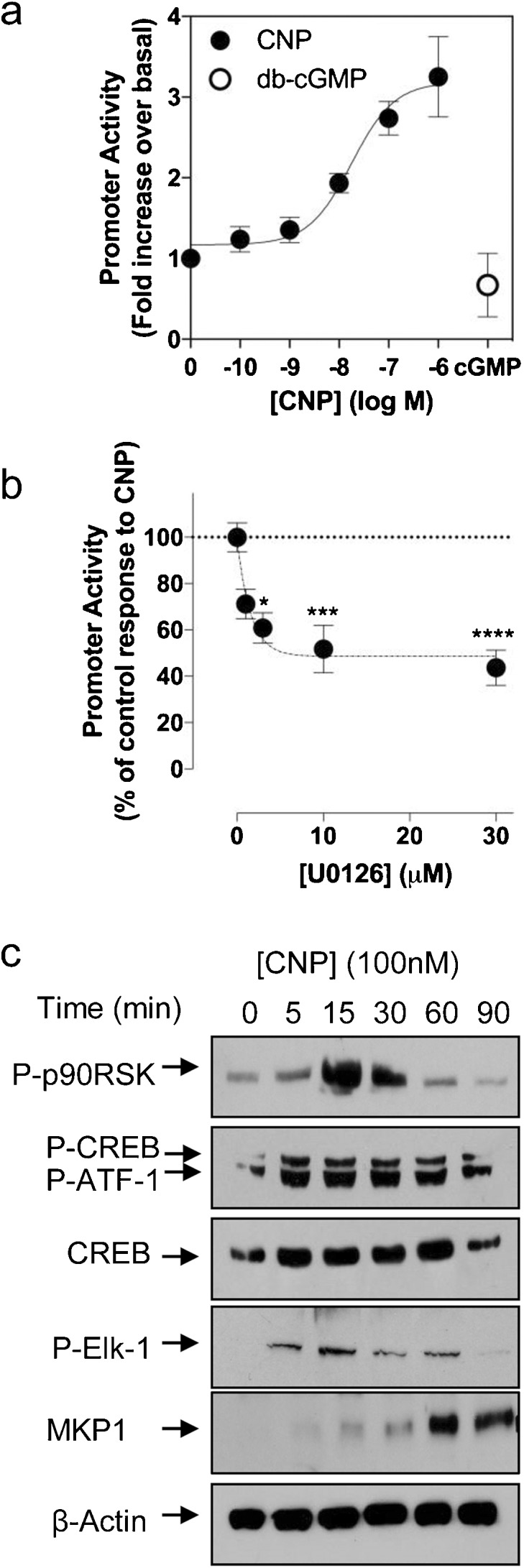
CNP stimulates αGSU promoter activity in a MEK-dependent manner. **a** GH3 cells were transiently transfected with 2.5 μg αGSU promoter and stimulated with the indicated concentrations of CNP or 1 mM db-cGMP. Cells were harvested after 8 h and protein extracts assayed for luciferase activity. Data shown are means ± SEM of three independent experiments, each performed in triplicate and are expressed as fold increase over basal. **b** GH3 cells transiently transfected with αGSU promoter (as above) were pre-treated with the indicated concentration of U0126 for 30 min prior to stimulation with CNP (100 nM). Cells were harvested after 8 h and protein extracts assayed for luciferase activity. Data shown are means ± SEM of three independent experiments, each performed in triplicate and are expressed as fold increase over control response to CNP (*P < 0.1, ***P < 0.001, ****P < 0.0001; significantly different from control response, as determined by Bonferonni’s multiple comparisons test). **c** CNP enhances phosphorylation of ERK1/2 pathway proteins. GH3 cells were stimulated with CNP (100 nM) for up to 90 min, prior to extraction of total proteins and Western blotting for the indicated target proteins. Each autoradiograph is representative of at least three independent experiments

Having excluded a proliferative effect of CNP in GH3 cells, we examined alternative biological consequences for CNP-stimulated P-ERK1/2 in GH3 cells. As immunofluorescence microscopy had identified the nuclear localisation of P-ERK1/2 in response to CNP (Fig. 2c–e’), we examined whether CNP-stimulated P-ERK1/2 was needed for gene transcription in GH3 cells, by examining the response of an ERK1/2-sensitive promoter, the human glycoprotein hormone α-subunit gene (αGSU), to CNP stimulation. GH3 cells were transiently transfected with the human αGSU promoter and stimulated for 8 h in the presence of CNP (0 to 1 μM). As shown (Fig. 6a), CNP caused a concentration-dependent increase in αGSU promoter activity (to 2.7 ± 0.2-fold and 3.3 ± 0.5-fold, *P* < 0.001 and *P* < 0.0001, for 100 nM and 1 μM CNP, respectively) but db-cGMP (1 mM) failed to have any effect. In parallel experiments, transiently transfected GH3 cells were pre-treated with the MEK inhibitor, U0126, for 30 min prior to stimulation with CNP and throughout the duration of the subsequent CNP stimulation. As shown (Fig. 6b), U0126 caused a concentration-dependent inhibition of CNP-stimulated αGSU promoter activity (to 60.7 ± 6.6%, 51.7 ± 10.3% and 43.7 ± 7.6%, *P* < 0.05, *P* < 0.001 and *P* < 0.0001, for 3 μM, 10 μM and 30 μM U0126, respectively). As P-ERK1/2 can target other transcriptional regulators for phosphorylation, we examined the phosphorylation status of p90-RSK, CREB, ATF1 and Elk-1 and the expression level of endogenous MKP1 in GH3 cells stimulated with 100 nM CNP. As shown (Fig. 6c), CNP treatment resulted in enhanced phosphorylation of each of the transcriptional regulators, together with the elevated expression of MKP1, over the duration of the time-course. Furthermore, as many stimuli can activate more than one member of the MAPK family, we assessed the ability of CNP to enhance p38 MAPK and JNK phosphorylation in GH3 cells. As shown (Supplemental Fig. 2), CNP enhanced the phosphorylation and nuclear localisation of both p38 MAPK and JNK in GH3 cells, an effect that was mirrored in the murine pituitary LβT2 cell line (Supplemental Fig. 3). These data suggest that gene transcription and transcriptional regulators are the biological targets for CNP-stimulated P-ERK1/2 in GH3 cells, rather than cell proliferation.

## Discussion

To date, biological effects mediated via the particulate guanylyl cyclases appear to require cGMP generation (Potter et al. 2006; Garbers et al. 2006). Despite the considerable evidence for non-cGMP-dependent effects of natriuretic peptides, these effects are mediated via the non-guanylyl cyclase, NPR-C receptor (Anand-Srivastava 2005), or by a renal-specific isoform (NPR-Bi) that fails to couple to cGMP (Hirsch et al. 2003). In the current study, we provide evidence of a cGMP-independent GC-B-dependent signalling event activated at concentrations lower than those required to initiate guanylyl cyclase activity.

Many studies have demonstrated the anti-proliferative effects of natriuretic peptides in the endothelial, bone and neuronal systems by mechanisms that predominantly involve the activation of the guanylyl cyclase/cGMP/PKG pathways (Chusho et al. 2001; Kaneki et al. 2008; Miyazawa et al. 2002; Krejci et al. 2005) or NPR-C activity (Gower et al. 2006). Our initial experiments reported here revealed the potent stimulation of ERK1/2 phosphorylation. Whereas the current observations were unexpected, given the previously reported inhibitory effects of natriuretic peptides on MAPK signalling (Hagiwara et al. 1996; Kaneki et al. 2008), other studies have reported stimulatory effects of natriuretic peptides on MAPK signalling in ventricular myocytes and in melanoma cells (Silberbach et al. 1999; Dhayade et al. 2016). However, these latter studies demonstrated a requirement for cGMP and PKG in mediating the ANP and CNP effects on ERK1/2 phosphorylation (Silberbach et al. 1999; Dhayade et al. 2016), whereas our own findings suggest GC-B-mediated ERK1/2 phosphorylation in GH3 cells is cGMP-independent. Specifically, pharmacological cGMP analogues (db-cGMP) failed to mimic the CNP effects on ERK1/2 phosphorylation in GH3 cells and enhanced ERK1/2 phosphorylation occurred at much lower concentrations of both CNP and ANP than are required to stimulate cGMP accumulation in these cells. Although we have not conclusively shown that cGMP signalling is uninvolved in the ERK response, cGMP signalling alone seems unlikely to be able to activate the ERK1/2 pathway in GH3 cells.

The kinetics and compartmentalisation of phosphorylated ERK1/2 can dictate the subsequent effects on downstream signalling events (Cobb et al. 1994; Caunt et al. 2006). Here, we show that CNP causes the enhanced phosphorylation of ERK1/2-associated proteins (p90RSK, CREB, ATF-1 and Elk-1). The enhanced phosphorylation of CREB, ATF-1 and Elk-1 might contribute to the pronounced effect that CNP elicits on the human αGSU promoter activity, which has been shown to be MEK-dependent. We have previously observed a robust response of the human αGSU promoter to CNP in LβT2 cells but not αT3–1 cells (Thompson et al. 2009), suggesting possible differences in the transcriptional regulation in these gonadotroph-derived cell lines. Whether the transcriptional response to CNP in LβT2 cells also requires MEK activity, as our current findings demonstrate in GH3 cells, remains to be determined. Furthermore, the concentration responses to CNP for cGMP accumulation, ERK1/2 phosphorylation and αGSU promoter activity interestingly do not overlap (although these responses cannot be directly compared, because of the differing sensitivities of each experimental approach). However, the potential combination of compartmentalised cGMP generation (for a review, see Arora et al. 2013) together with the activation of MEK signalling might contribute to shaping the biological responsiveness to CNP. Such an interaction of cGMP and MEK pathways has been previously demonstrated in osteoblasts (Rangaswami et al. 2009). Finally, our findings that CNP also potently activates the JNK and p38 MAPK pathways in GH3 cells add further complexity to GC-B down-stream signalling. Whereas our data do not identify putative upstream kinases that might explain this triple activation of MAPK pathways in GH3 cells, cross-activation of p38 MAPK and JNK might occur via ERK1/2, as reviewed by others (Zhang and Liu 2002; Roux and Blenis 2004; McKay and Morrison 2007).

Despite the prolonged nature of the ERK1/2 response to CNP, GH3 cells failed to proliferate following a CNP challenge. In many other systems, CNP causes anti-proliferative effects, predominantly via cGMP-dependent pathways (Chusho et al. 2001; Kaneki et al. 2008; Miyazawa et al. 2002; Krejci et al. 2005). Although these effects are no doubt cell-type-specific, dual activation of ERK1/2 phosphorylation and cGMP production in GH3 cells might nullify any potential anti-proliferative effect of cGMP. However, whereas our current investigations used a comprehensive range of cell proliferation techniques, such data have to be interpreted with caution when using tumour-derived cells lines, such as GH3 cells (Tashjian et al. 1968; Faivre-Bauman et al. 1975). The potential effects of CNP on primary pituitary cell proliferation and on ERK1/2 signalling remain to be elucidated.

Given the broad range of GC-A, GC-B and NPR-C expression, most cell types are likely to express at least one of these mammalian natriuretic peptide receptors (Potter et al. 2006). Non-cGMP-mediated effects of natriuretic peptides are usually attributed to NPR-C activity (Anand-Srivastava 2005); however, our current studies failed to demonstrate a role for NPR-C in GH3 cells. Limited reports have suggested that splice variants and isoforms of the GC-B receptor exist in mice and humans. At least three splice variants (GC-B1, 2 and 3) are expressed in mice, whereby GC-B1 and GC-B2 have guanylyl cyclase activity, although the partially truncated GC-B2 is no longer hormone-sensitive (Tamura and Garbers 2003). GC-B3 is severely truncated and appears not to mediate down-stream signalling events (Tamura and Garbers 2003). In human proximal tubules, the NprBi splice variant regulates potassium channels in a non-cGMP- but tyrosine kinase-dependent manner (Hirsch et al. 2003) as demonstrated through genistein sensitivity. Importantly, CNP failed to stimulate cGMP production via the NPR-Bi (Hirsch et al. 2003), whereas our current data show CNP to stimulate both cGMP and ERK1/2 phosphorylation in GH3 cells. Furthermore, we found that the rat GH3 cell line also expresses all three splice variants described in mice and that GC-B1 and GC-B2 are lost following siRNA targeting. Therefore, one or both of these splice variants might mediate the CNP effect on ERK1/2 signalling. However, the lack of effect of numerous receptor-tyrosine kinase inhibitors (including genistein), coupled to an intact cGMP response to CNP in GH3 cells, suggests that a rat homologue of NprBi is not involved.

GC-B signalling has been shown to be sensitive to changes in intracellular calcium and PKC activity in many cell types (Potter et al. 2006; Potter and Hunter 2000; Abbey and Potter 2002; Abbey-Hosch et al. 2005). Elevated calcium concentrations or enhanced PKC activity have been shown to cause heterologous desensitisation of GC-B-mediated cGMP accumulation (Potthast et al. 2004; Fowkes et al. 2000; Thompson et al. 2014) and both of these pathways mediate ERK1/2 activation in pituitary cell lines (Mulvaney and Roberson 2000; Naor et al. 2000). Our studies suggest that CNP-stimulated ERK1/2 phosphorylation does not require Ca^2+^ or PKC activity but does need MEK1/2 activity and the partial activation of a non-receptor tyrosine kinase (possibly Src kinase), as determined by PP2 treatments. Particulate guanylyl cyclase receptors are structurally similar to tyrosine kinase receptors; the GC-B is not uncommon in this regard, itself harbouring a tyrosine-kinase-like domain. Conventional receptor tyrosine kinases can activate the MAPK pathways via the Raf and MEK pathways (McKay and Morrison 2007) but particulate guanylyl cyclases have not yet been demonstrated to exert tyrosine kinase activity. *In silico* examination of the intracellular domains of the GC-B receptor failed to identify SH-2 domains and, thorough proteomic investigations of putative phosphorylation sites, did not identify tyrosine residues (Potter and Hunter 1998). Thus, the exact nature of the upstream participants that mediate GC-B-dependent ERK1/2 phosphorylation remains enigmatic. However, other studies have revealed the recruitment of particulate guanylyl cyclases by the small GTPase proteins, Rac and PAK (Guo et al. 2007). In their study, PAK was shown to activate particulate guanylyl cyclases directly in order to stimulate modest cGMP accumulation (Guo et al. 2007) through a protein-protein interaction. Our current data intriguingly juxtapose these findings and suggest that a complimentary relationship exists between growth factor receptor signalling and GC-B.

Particulate guanylyl cyclases are critical in mediating a wide-range of homeostatic processes but these cannot be explained solely by cGMP generation. Our work suggests an intriguing additional mechanism by which conventional GC-B receptors can signal, without the requirement for cGMP and hints at novel biological roles for these receptors in a range of tissues.

## Electronic supplementary material

Supplemental Figure 1 Calcium, PKA, PKC and PI3K pathways do not mediate CNP-stimulated ERK1/2 phosphorylation in GH3 cells. GH3 cells were pre-treated for 30 min (15 min for nifedipine) with various concentrations (0, 1, 3, 10, 30 μM) of BAPTA-AM, H-89, nifedipine, GF109203X or PI3K inhibitor (LY294006) before stimulation with 100 nM CNP and subsequent Western blotting for ERK1/2 phosphorylation. Each autoradiograph is representative of two independent experiments. (GIF 30 kb)

High Resolution Image (TIFF 729 kb)

Supplemental Figure 2 CNP stimulates phosphorylation of p38-MAPK and JNK in GH3 cells. **a** GH3 cells were treated for up to 90 min with 100 nM CNP prior to being fixed and stained for phospho-p38 MAPK (*left*) or phospho-JNK (*right*; Alexa-488, *green*) or nuclear co-staining (DAPI, *blue*). Immunofluorescence was visualised by using confocal microscopy. Images shown are a representative field of vision from two independent experiments. **b** GH3 cells were stimulated with CNP (100 nM) for up to 90 min prior to extraction of total proteins and Western blotting for phospho-p38 MAPK. **c** GH3 cells were stimulated for 15 min with the indicated concentrations of CNP prior to extraction of total proteins for Western blotting for phospho-JNK. Each autoradiograph is representative of two independent experiments. (GIF 48 kb)

High Resolution Image (TIFF 955 kb)

Supplemental Figure 3 CNP enhances phosphorylation of ERK1/2 pathway proteins in mouse LβT2 gonadotrope cells. LβT2 cells were stimulated with CNP (100 nM) for up to 90 min prior to extraction of total proteins and Western blotting for the indicated target proteins. Each autoradiograph is representative of two independent experiments. (GIF 17 kb)

High Resolution Image (TIFF 509 kb)

## References

[CR1] Abbey SE, Potter LR (2002). Vasopressin-dependent inhibition of the C-type natriuretic peptide receptor, NPR-B/GC-B, requires elevated intracellular calcium concentrations. J Biol Chem.

[CR2] Abbey-Hosch SE, Smirnov D, Potter LR (2005). Differential regulation of NPR-B/GC-B by protein kinase c and calcium. Biochem Pharmacol.

[CR3] Anand-Srivastava MB (2005). Natriuretic peptide receptor-C signaling and regulation. Peptides.

[CR4] Arora K, Sinha C, Zhang W, Ren A, Moon CS, Yarlagadda S, Naren AP (2013). Compartmentalization of cyclic nucleotide signaling: a question of when, where, and why?. Pflugers Arch.

[CR5] Caunt CJ, Finch AR, Sedgley KR, McArdle CA (2006). Seven-transmembrane receptor signalling and ERK compartmentalization. Trends Endocrinol Metab.

[CR6] Chen G, Zhao J, Yin Y, Wang B, Liu Q, Li P, Zhao L, Zhou H (2014). C-type natriuretic peptide attenuates LPS-induced endothelial activation: involvement of p38, Akt, and NF-κB pathways. Amino Acids.

[CR7] Chusho H, Tamura N, Ogawa Y, Yasoda A, Suda M, Miyazawa T, Nakamura K, Nakao K, Kurihara T, Komatsu Y, Itoh H, Tanaka K, Saito Y, Katsuki M, Nakao K (2001). Dwarfism and early death in mice lacking C-type natriuretic peptide. Proc Natl Acad Sci U S A.

[CR8] Cobb MH, Hepler JE, Cheng M, Robbins D (1994). The mitogen-activated protein kinases, ERK1 and ERK2. Semin Cancer Biol.

[CR9] De Jonge HR, Tilly BC, Hogema BM, Pfau DJ, Kelley CA, Kelley MH, Melita AM, Morris MT, Viola RM, Forrest JN (2014). cGMP inhibition of type 3 phosphodiesterase is the major mechanism by which C-type natriuretic peptide activates CFTR in the shark rectal gland. Am J Physiol Cell Physiol.

[CR10] Dhayade S, Kaesler S, Sinnberg T, Dobrowinski H, Peters S, Naumann U, Liu H, Hunger RE, Thunemann M, Biedermann T, Schittek B, Simon HU, Feil S, Feil R (2016). Sildenafil potentiates a cGMP-dependent pathway to promote melanoma growth. Cell Rep.

[CR11] Faivre-Bauman A, Gourdji D, Grouselle D, Tixier-Vidal A (1975). Binding of thyrotropin releasing hormone and prolactin release by synchronized GH3 rat pituitary cell line. Biochem Biophys Res Commun.

[CR12] Fowkes RC, Forrest-Owen W, Williams B, McArdle CA (1999). C-type natriuretic peptide (CNP) effects on intracellular calcium [Ca2+]i in mouse gonadotrope-derived αT3-1 cell line. Regul Pept.

[CR13] Fowkes RC, McArdle CA (2000). C-type natriuretic peptide: an important neuroendocrine regulator?. Trends Endocrinol Metab.

[CR14] Fowkes RC, Forrest-Owen W, McArdle CA (2000). C-type natriuretic peptide (CNP) effects in anterior pituitary cell lines: evidence for homologous desensitisation of CNP-stimulated cGMP accumulation in αT3-1 gonadotroph-derived cells. J Endocrinol.

[CR15] Garbers DL, Chrisman TD, Wiegn P, Katafuchi T, Albanesi JP, Bielinski V, Barylko B, Redfield MM, Burnett JC (2006). Membrane guanylyl cyclase receptors: an update. Trends Endocrinol Metab.

[CR16] Gower WR, Carter GM, McAfee Q, Solivan SM (2006). Identification, regulation and anti-proliferative role of the NPR-C receptor in gastric epithelial cells. Mol Cell Biochem.

[CR17] Grandclément B, Brisson C, Bayard F, Tremblay J, Gossard F, Morel G (1995). Localization of mRNA coding for the three subtypes of atrial natriuretic factor (ANF) receptors in rat anterior pituitary gland cells. J Neuroendocrinol.

[CR18] Guo D, Tan YC, Wang D, Madhusoodanan KS, Zheng Y, Maack T, Zhang JJ, Huang XY (2007). A Rac-cGMP signaling pathway. Cell.

[CR19] Hagiwara H, Inoue A, Furuya M, Tanaka S, Hirose S (1996). Change in the expression of C-type natriuretic peptide and its receptor, B-type natriuretic peptide receptor, during dedifferentiation of chondrocytes into fibroblast-like cells. J Biochem.

[CR20] Hartt DJ, Ogiwara T, Ho AK, Chik CL (1995). Cyclic GMP stimulates growth hormone release in rat anterior pituitary cells. Biochem Biophys Res Commun.

[CR21] Hirsch JR, Skutta N, Schlatter E (2003). Signaling and distribution of NPR-bi, the human splice form of the natriuretic peptide receptor type B. Am J Physiol Renal Physiol.

[CR22] Kaneki H, Kurokawa M, Ide H (2008). The receptor attributable to C-type natriuretic peptide-induced differentiation of osteoblasts is switched from type B- to type C-natriuretic peptide receptor with aging. J Cell Biochem.

[CR23] Krejci P, Masri B, Fontaine V, Mekikian PB, Weis M, Prats H, Wilcox WR (2005). Interaction of fibroblast growth factor and C-natriuretic peptide signaling in regulation of chondrocyte proliferation and extracellular matrix homeostasis. J Cell Sci.

[CR24] Komatsu Y, Nakao K, Suga S, Ogawa Y, Mukoyama M, Arai H, Shirakami G, Hosoda K, Nakagawa O, Hama N (1991). C-type natriuretic peptide (CNP) in rats and humans. Endocrinology.

[CR25] McArdle CA, Poch A, Käppler K (1993). Cyclic guanosine monophosphate production in the pituitary: stimulation by C-type natriuretic peptide and inhibition by gonadotropin-releasing hormone in αT3-1 cells. Endocrinology.

[CR26] McKay MM, Morrison DK (2007). Integrating signals from RTKs to ERK/MAPK. Oncogene.

[CR27] Miyazawa T, Ogawa Y, Chusho H, Yasoda A, Tamura N, Komatsu Y, Pfeifer A, Hofmann F, Nakao K (2002). Cyclic GMP-dependent protein kinase II plays a critical role in C-type natriuretic peptide-mediated endochondral ossification. Endocrinology.

[CR28] Mulvaney JM, Roberson MS (2000). Divergent signaling pathways requiring discrete calcium signals mediate concurrent activation of two mitogen-activated protein kinases by gonadotropin-releasing hormone. J Biol Chem.

[CR29] Naor Z, Benard O, Seger R (2000). Activation of MAPK cascades by G-protein-coupled receptors: the case of gonadotropin-releasing hormone receptor. Trends Endocrinol Metab.

[CR30] Potter LR, Hunter T (1998). Identification and characterization of the major phosphorylation sites of the B-type natriuretic peptide receptor. J Biol Chem.

[CR31] Potter LR, Hunter T (2000). Activation of protein kinase C stimulates the dephosphorylation of natriuretic peptide receptor-B at a single serine residue: a possible mechanism of heterologous desensitization. J Biol Chem.

[CR32] Potter LR, Abbey-Hosch S, Dickey DM (2006). Natriuretic peptides, their receptors, and cyclic guanosine monophosphate-dependent signaling functions. Endocr Rev.

[CR33] Potthast R, Abbey-Hosch SE, Antos LK, Marchant JS, Kuhn M, Potter LR (2004). Calcium-dependent dephosphorylation mediates the hyperosmotic and lysophosphatidic acid-dependent inhibition of natriuretic peptide receptor-B/guanylyl cyclase-B. J Biol Chem.

[CR34] Rangaswami H, Marathe N, Zhuang S, Chen Y, Yeh JC, Frangos JA, Boss GR, Pilz RB (2009). Type II cGMP-dependent protein kinase mediates osteoblast mechanotransduction. J Biol Chem.

[CR35] Rose RA, Hatano N, Ohya S, Imaizumi Y, Giles WR (2007). C-type natriuretic peptide activates a non-selective cation current in acutely isolated rat cardiac fibroblasts via natriuretic peptide C receptor-mediated signalling. J Physiol (Lond).

[CR36] Roux PP, Blenis J (2004). ERK and p38 MAPK-activated protein kinases: a family of protein kinases with diverse biological functions. Microbiol Mol Biol Rev.

[CR37] Sciarretta S, Marchitti S, Bianchi F, Moyes A, Barbato E, Di Castro S, Stanzione R, Cotugno M, Castello L, Calvieri C, Eberini I, Sadoshima J, Hobbs AJ, Volpe M, Rubattu S (2013). C2238 atrial natriuretic peptide molecular variant is associated with endothelial damage and dysfunction through natriuretic peptide receptor C signaling. Circ Res.

[CR38] Shimekake Y, Ohta S, Nagata K (1994). C-type natriuretic peptide stimulates secretion of growth hormone from rat-pituitary-derived GH3 cells via a cyclic-GMP-mediated pathway. Eur J Biochem.

[CR39] Silberbach M, Gorenc T, Hershberger RE, Stork PJ, Steyger PS, Roberts CT (1999). Extracellular signal-regulated protein kinase activation is required for the anti-hypertrophic effect of atrial natriuretic factor in neonatal rat ventricular myocytes. J Biol Chem.

[CR40] Sun JB, Huang X, Xu HY, Li XL, Gao L, Kim YC, Xu WX (2006). Inhibitory effect of C-type natriuretic peptide on L-type calcium channel currents in gastric antral myocytes of guinea pigs. Gen Physiol Biophys.

[CR41] Tamura N, Garbers DL (2003). Regulation of the guanylyl cyclase-B receptor by alternative splicing. J Biol Chem.

[CR42] Tamura N, Doolittle LK, Hammer RE, Shelton JM, Richardson JA, Garbers DL (2004). Critical roles of the guanylyl cyclase B receptor in endochondral ossification and development of female reproductive organs. Proc Natl Acad Sci U S A.

[CR43] Tashjian AH, Yasumura Y, Levine L, Sato GH, Parker ML (1968). Establishment of clonal strains of rat pituitary tumor cells that secrete growth hormone. Endocrinology.

[CR44] Thompson IR, Chand AN, Jonas KC, Burrin JM, Steinhelper ME, Wheeler-Jones CPD, McArdle CA, Fowkes RC (2009). Molecular characterization and functional interrogation of a local natriuretic peptide system in rodent pituitaries, αT3-1 and LβT2 gonadotrophs. J Endocrinol.

[CR45] Thompson IR, Chand AN, King PJ, Ansorge O, Karavitaki N, Jones CA, Rahmutula D, Gardner DG, Zivkovic V, Wheeler-Jones CP, McGonnell IM, Korbonits M, Anderson RA, Wass JA, McNeilly AS, Fowkes RC (2012). Expression and transcriptional regulation of guanylyl cyclase-B (GC-B) receptors in a range of human pituitary adenomas, normal human fetal pituitaries and anterior pituitary cell lines. Endocr Rel Cancer.

[CR46] Thompson IR, Mirczuk SM, Smith L, Lessey AJ, Simbi B, Sunters A, Baxter GF, Lipscomb VJ, McGonnell IM, Wheeler-Jones CP, Mukherjee A, McArdle CA, Roberson MS, Fowkes RC (2014). Molecular and pharmacological characterization of particulate guanylyl cyclases in GH3 somatolactotropes: evidence for common and distinct regulation by atrial and C-type natriuretic peptides. Cell Tissue Res.

[CR47] Zhang W, Liu HT (2002). MAPK signal pathways in the regulation of cell proliferation in mammalian cells. Cell Res.

[CR48] Zenitani M, Nojiri T, Uehara S, Miura K, Hosoda H, Kimura T, Nakahata K, Miyazato M, Okuyama H, Kangawa K (2016). C-type natriuretic peptide in combination with sildenafil attenuates proliferation of rhabdomyosarcoma cells. Cancer Med.

