# Supplementary figures and images for: Natriuretic peptide activation of extracellular regulated kinase 1/2 (ERK1/2) pathway by particulate guanylyl cyclases in GH3 somatolactotropes
Source: Cell Tissue Res. 2017 Apr 27;369(3):567–78. doi: 10.1007/s00441-017-2624-x (PMC5579180; doi:10.1007/s00441-017-2624-x)

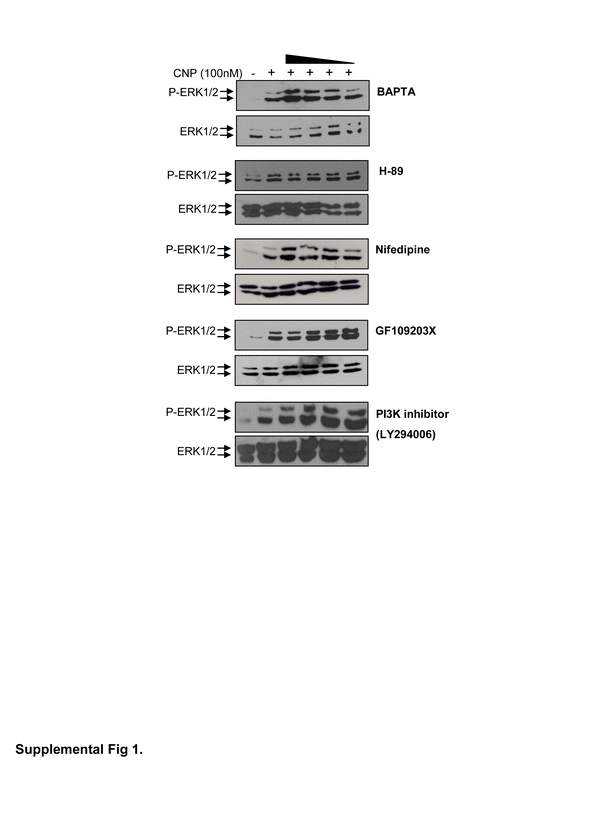

Supplement: Supplementary file 1 — Calcium, PKA, PKC and PI3K pathways do not mediate CNP-stimulated ERK1/2 phosphorylation in GH3 cells. GH3 cells were pre-treated for 30 min (15 min for nifedipine) with various concentrations (0, 1, 3, 10, 30 μM) of BAPTA-AM, H-89, nifedipine, GF109203X or PI3K inhibitor (LY294006) before stimulation with 100 nM CNP and subsequent Western blotting for ERK1/2 phosphorylation. Each autoradiograph is representative of two independent experiments. (GIF 30 kb) [file 441_2017_2624_Fig7_ESM.gif]

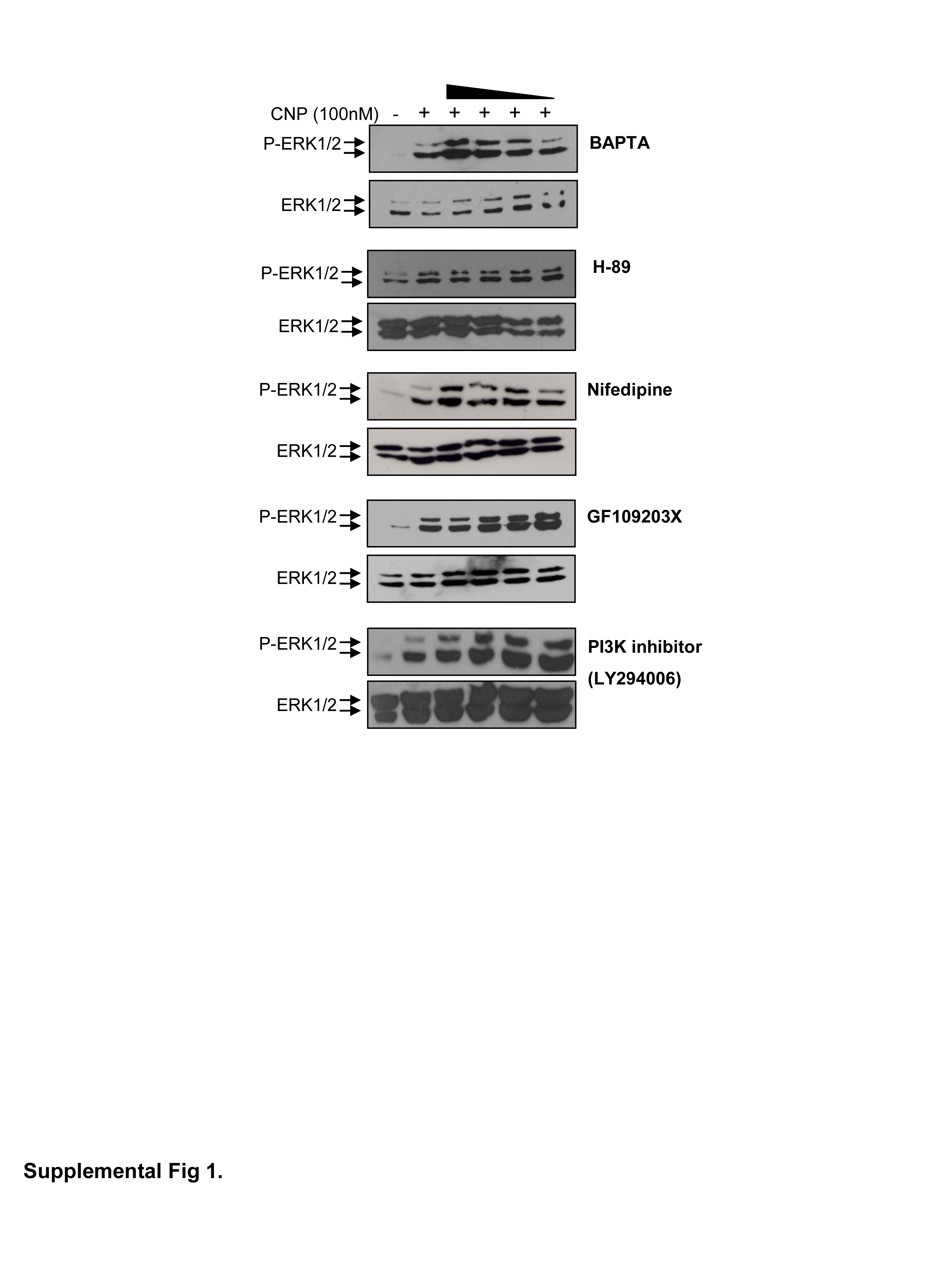

Supplement: Supplementary file 2 — High Resolution Image (TIFF 729 kb) [file 441_2017_2624_MOESM1_ESM.tif]

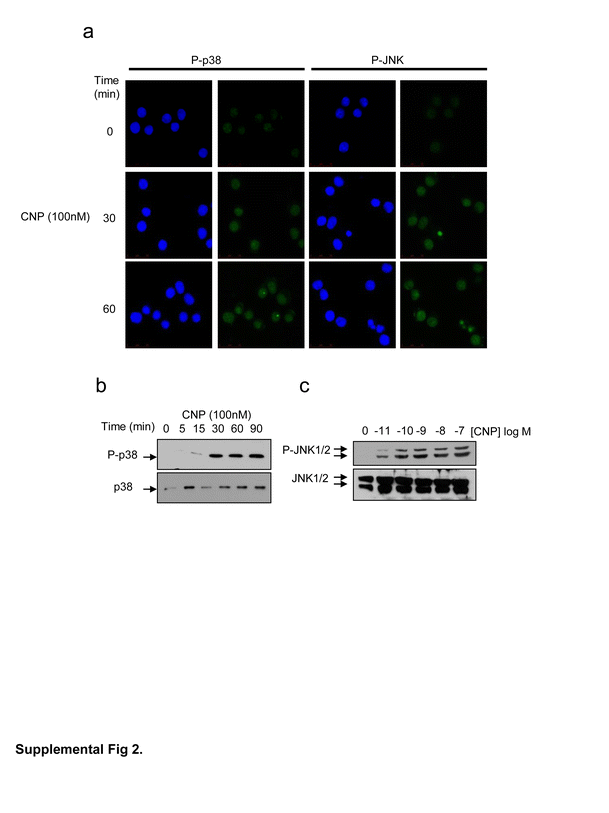

Supplement: Supplementary file 3 — CNP stimulates phosphorylation of p38-MAPK and JNK in GH3 cells. a GH3 cells were treated for up to 90 min with 100 nM CNP prior to being fixed and stained for phospho-p38 MAPK (left) or phospho-JNK (right; Alexa-488, green) or nuclear co-staining (DAPI, blue). Immunofluorescence was visualised by using confocal microscopy. Images shown are a representative field of vision from two independent experiments. b GH3 cells were stimulated with CNP (100 nM) for up to 90 min prior to extraction of total proteins and Western blotting for phospho-p38 MAPK. c GH3 cells were stimulated for 15 min with the indicated concentrations of CNP prior to extraction of total proteins for Western blotting for phospho-JNK. Each autoradiograph is representative of two independent experiments. (GIF 48 kb) [file 441_2017_2624_Fig8_ESM.gif]

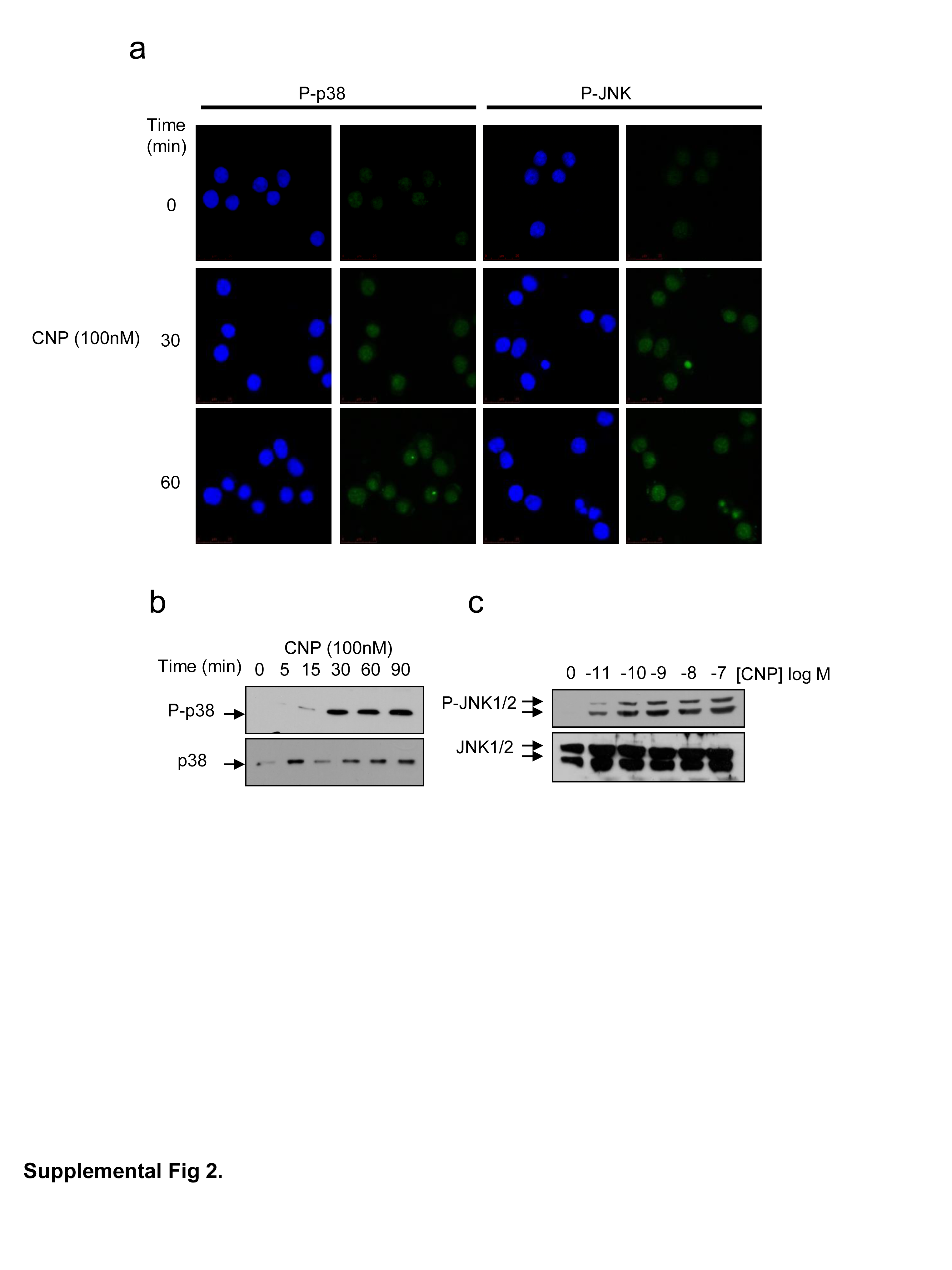

Supplement: Supplementary file 4 — High Resolution Image (TIFF 955 kb) [file 441_2017_2624_MOESM2_ESM.tif]

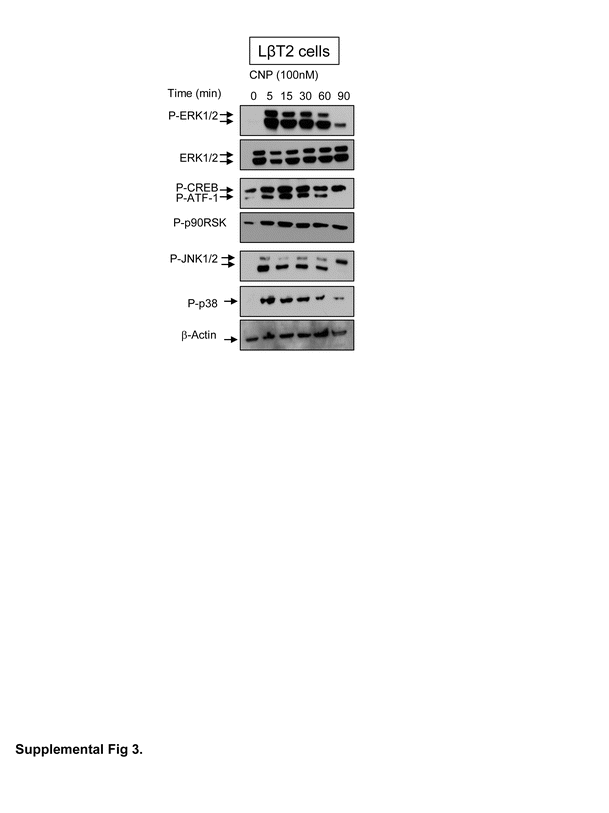

Supplement: Supplementary file 5 — CNP enhances phosphorylation of ERK1/2 pathway proteins in mouse LβT2 gonadotrope cells. LβT2 cells were stimulated with CNP (100 nM) for up to 90 min prior to extraction of total proteins and Western blotting for the indicated target proteins. Each autoradiograph is representative of two independent experiments. (GIF 17 kb) [file 441_2017_2624_Fig9_ESM.gif]

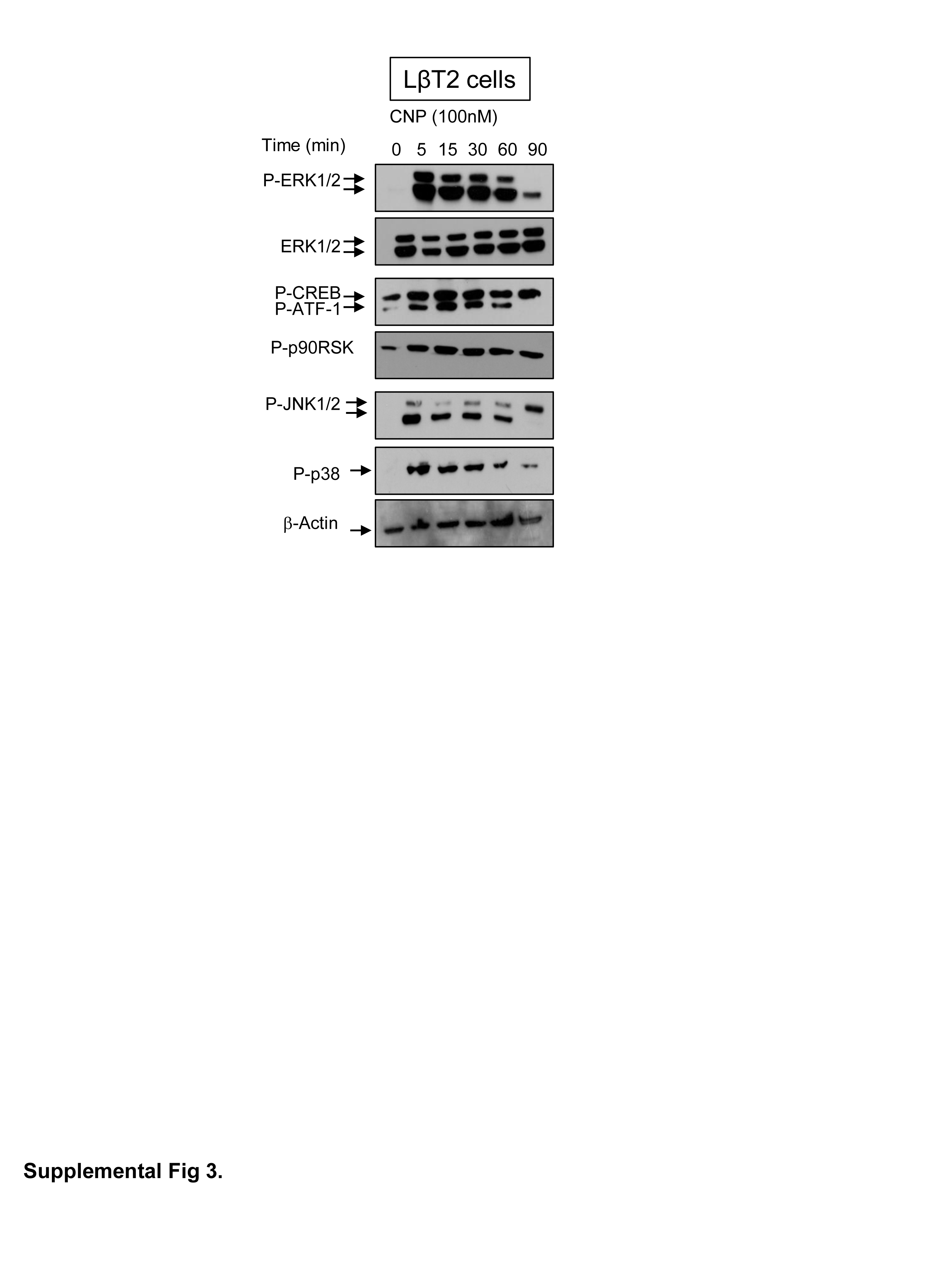

Supplement: Supplementary file 6 — High Resolution Image (TIFF 509 kb) [file 441_2017_2624_MOESM3_ESM.tif]
